# Exploring the Interplay of the CRISPR-CAS System with Antibiotic Resistance in *Staphylococcus aureus*: A Poultry Meat Study from Lahore, Pakistan

**DOI:** 10.3390/medicina60010130

**Published:** 2024-01-10

**Authors:** Muhammad Abu Bakr Shabbir, Aziz Ul-Rahman, Muhammad Rizwan Iftikhar, Majeeda Rasheed, Muhammad Kashif Maan, Adeel Sattar, Mehmood Ahmad, Farid Ahmed Khan, Waqas Ahmad, Muhammad Ilyas Riaz, Hassaan Bin Aslam

**Affiliations:** 1 Institute of Microbiology, Faculty of Veterinary Sciences, University of Veterinary and Animal Sciences, Lahore 54000, Pakistanfaridahmedkhan@hotmail.com (F.A.K.);; 2 Department of Pathobiology and Biomedical Sciences, Faculty of Veterinary and Animal Sciences, Muhammad Nawaz Shareef (MNS) University of Agriculture, Multan 66000, Pakistan; aziz.rahman@mnsuam.edu.pk; 3 Department of life Sciences, Khwaja Fareed University of Engineering and Information Technology, Rahim Yar Khan 64200, Pakistan; majeeda.rasheed@kfueit.edu.pk; 4 Department of Veterinary Surgery, Faculty of Veterinary Sciences, University of Veterinary and Animal Sciences, Lahore 54000, Pakistan; 5 Department of Pharmacology and Toxicology, Faculty of Biosciences, University of Veterinary and Animal Sciences, Lahore 54000, Pakistan; 6 Department of Pharmacology and Toxicology, Faculty of Veterinary and Animal Sciences, The Islamia University of Bahawalpur, Bahawalpur 63100, Pakistan; mehmood.ahmad@iub.edu.pk; 7 Department of Pathology, Faculty of Veterinary Sciences, University of Veterinary and Animal Sciences, Lahore 54000, Pakistan; waqasahmadvet@gmail.com

**Keywords:** *S. aureus*, CRISPR-cas system, *Cas10* gene, MDR

## Abstract

*Staphylococcus aureus* is one of the major pathogens responsible for causing food poisoning worldwide. The emergence of antibiotic resistance in this bacterium is influenced by various factors. Among them, bacterial acquired defense systems described as clustered regularly interspaced short palindromic repeats (CRISPR)-cas system might be involved in antibiotic resistance development in bacteria. The current study was designed to assess the prevalence of *S. aureus* and its antibiotic resistance profile and identify the relationship of the CRISPR-cas system with antimicrobial resistance, followed by phylogenetic analysis. Total samples (*n* = 188) of poultry meat were collected from the poultry bird market of Lahore, Punjab, Pakistan. We used both phenotypic (antibiotic disc diffusion) and genotypic methods (PCR) to identify multi-drug resistant (MDR) strains of S. aureus. Additionally, the role of the CRISPR-Cas system in the isolated MDR S. aureus was also assessed. In addition, real-time quantitative PCR (qRT-PCR) was used to evaluate the association of the CRISPR-cas system with antimicrobial resistance. All of the *S. aureus* isolates showed 100% resistance against erythromycin, 97.5% were resistant to tetracycline, and 75% were resistant to methicillin. Eleven isolates were MDR in the current study. The CRISPR system was found in all MDR isolates, and fifteen spacers were identified within the CRISPR locus. Furthermore, MDR *S. aureus* isolates and the standard strain showed higher expression levels of CRISPR-associated genes. The correlation of said system with MDR isolates points to foreign gene acquisition by horizontal transfer. Current knowledge could be utilized to tackle antibiotic-resistant bacteria, mainly *S. aureus*.

## 1. Introduction

The poultry sector is a crucial segment of Pakistan’s agriculture system [1]. Chicken meat is an excellent source of protein, appealing to consumers because of its sensorial qualities [2]. Despite rapid growth in this industry, infectious diseases have posed a risk at a large scale [3]. One of the most frequent causes of bone infections in chickens is *staphylococcus*, which is also one of the most historically documented bacterial diseases in poultry [4].

*S. aureus* is a leading cause of foodborne illness, responsible for 241,000 illnesses yearly in the United States [2]. Recently, it has been documented that methicillin-resistant *S. aureus* (MRSA) was detected in 49% of all reported cases in Pakistan [5]. This bacterium is a typical inhabitant as commensal flora in humans and livestock [6]. *S. aureus* is responsible for causing infection in animals (mastitis) as well as humans (endocarditis and hemolytic pneumonia) [7]. This bacterium is responsible for various bird conditions, including omphalitis, synovitis, arthritis, and septicemia [6]. These diseases affect a massive proportion of poultry flocks [8]. *S. aureus* originating from livestock is transferred to humans via direct interaction with animals or through contaminated food [9], which causes the production of enterotoxins, resulting in gastroenteritis, dermal infection, and respiratory infection in consumers [6].

Antimicrobial resistance (AMR) in bacteria is a natural phenomenon [10], considered to be a significant health threat, and the emergence of antibiotic-resistant bacteria needs up-to-date treatment protocols [11,12]. According to a reported economic study, the number of fatalities associated with antibiotic-resistant microbes will increase exponentially from 700,000 per year in 2015 to 10 million per year in 2050 [13]. This shows that resistant microbes will cause a higher death rate in comparison to that of cancer, road accidents, and diabetes [14].

*S. aureus* is receiving wider attention because of antibiotic resistance; one of the resistant strains is methicillin-resistant *Staphylococcus aureus* (MRSA). MRSA is an alarming situation for humans as it has the potential for zoonotic transmission [2]. Resistance to antimicrobials develops because of irrational usage, mutations, clonal evolution, or plasmid transfer. It has been conclusively shown that *Staphylococcus* gain antimicrobial resistance genes (ARGs) through mobile genetic elements (MGEs), such as plasmids [15,16], and horizontal gene transfer (HGT) allows the dissemination of ARGs across the bacterial population [17].

The CRISPR-cas system is determined to be a bacterial defense system that prevents invasion by foreign genes. The architecture of that system consists of a CRISPR array and 6–20 adjacent genes [14]. The CRISPR array is composed of a small direct repeat (DR) sequence of 21–48 base pairs (bps) separated by a 26–72 bp spacer sequence [18]. This system acts via three stages: adaptation, biogenesis, and interference. CRISPR recognizes and blocks the transfer of MGEs that resemble the CRISPR spacer sequence [19]. Based on the signature gene, this system is divided into three types: I, II, and III [14].

*S. aureus* has a type III CRISPR-cas system [18] that consists of cas proteins like Cas2, Cas1, Cas6, Cas10, csm3, csm2, csm6, csm4, and csm5 [20]. Csm is a type III-A CRISPR-cas interference complex. Cas2 and Cas1 are involved in spacer acquisition. Cas6 is necessary for initial crRNA processing, while csm1–5 share a csm effector complex that directly interferes with the targeted sequence [18].

Researchers in 2014 reported that this system enhances envelope integrity in *Francisella novicida* via bacterial lipoprotein regulation and consequently conferring resistance to diverse membrane stressors, including antibiotics [21]. Similarly, it was reported that the CRISPR-cas system maintains genetic homeostasis by defending the host genome against invaders [22]. However, several studies have identified an inverse correlation of the CRISPR-cas system with the presence of plasmids and phages, as illustrated in *Enterococcus*, *Campylobacter*, and various species within Group A *Streptococcus* [23].

Apart from its function as a defensive mechanism, the CRISPR-cas system’s functioning in antimicrobial resistance is under debate [24]. Therefore, the current study aimed to find the CRISPR-cas system in MDR *S. aureus* and its association with MDR *S. aureus* isolated from chicken meat in Lahore. It is believed that the present study’s findings will provide a basis for understanding the CRISPR-cas system’s association with antibiotic resistance and help us further study how this regulates AMR in said bacteria.

## 2. Results

### 2.1. Bacterial Isolates Conformation

**Figure 1:**
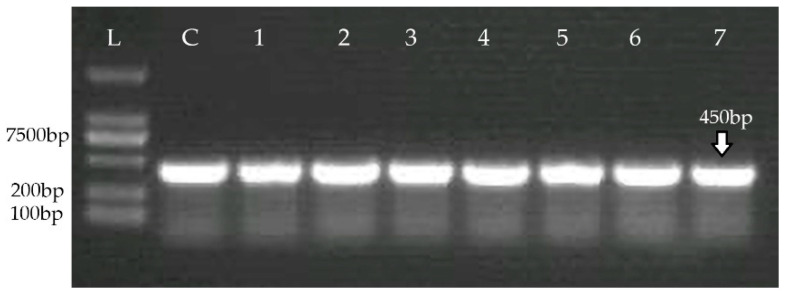
Molecular confirmation of *S. aureus* isolates. L, DNA ladder; C, positive control. Numerals represent the number of different *S. aureus* isolates.

In total, 188 swab samples of chicken meat were collected, cultured, and processed via standard conventional bacteriological methods and PCR. Of the 188 collected samples, 38.2% were confirmed as *Staphylococcus*, and 21% were molecularly confirmed as *S. aureus* using PCR. Samples were biochemically identified as *S. aureus*. Of these 73 samples, 54.79% (40/73) of isolates were identified as *S. aureus* via PCR (Figure 1). 

### 2.2. Antibiotic Resistance Pattern

The antibiotic resistance pattern of all 40 confirmed isolates of *S. aureus* against seven antibiotics was determined. *S. aureus* isolates showed maximum resistance to erythromycin (E) (100%), tetracycline (TE) (97.5%), methicillin (75%), and vancomycin (VAN) (55%) followed by an intermediate level of resistance to ciprofloxacin (CIP) (50%), and chloramphenicol (CHL) (42.5%) and a lower level of resistance against gentamicin, as represented in Table 1.

### 2.3. Detection of Antibiotic Resistance Genes

**Figure 2:**
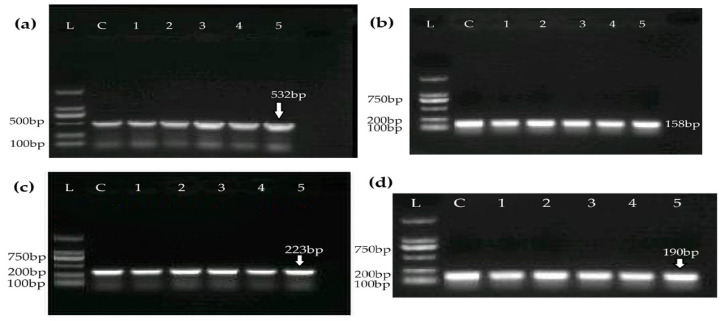
Identification of ARGs via PCR: (**a**) *mecA*, (**b**) *tetM*, (**c**) *gyrA*, and (**d**) *ermA*. L, DNA ladder; C, positive control. Numerals represent the number of different *S. aureus* isolates.

PCR was employed to evaluate four AMR genes (*mecA, tetM, gyrA,* and *ermA*) among all confirmed *S. aureus* isolates; the results are presented in Figure 2. In total, 11 out of 40 isolates were found to be MDR. Moreover, one or two of these AMR genes were identified among the remaining isolates.

### 2.4. Detection of CRISPR-Cas System

**Figure 3:**
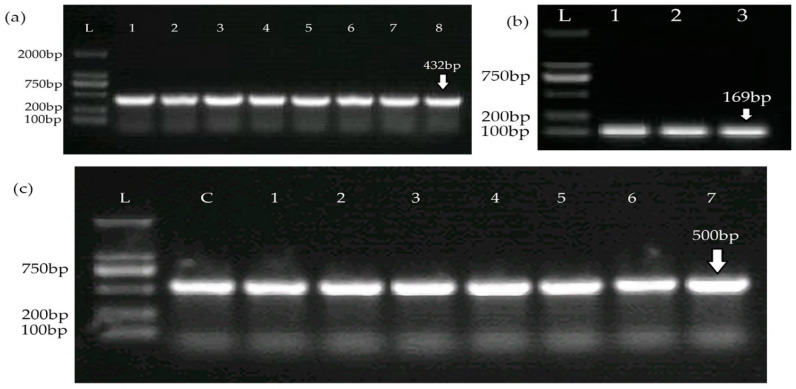
Detection of the CRISPR Cas system type III: (**a**) The presence of the *Cas1* gene; numericals 1–8 represent different *S. aureus* isolates. (**b**) The presence of the *Cas2* gene; numericals 1–3 represent different *S. aureus* isolates. (**c**) The presence of the *Cas10* gene; numericals 1–7 represent different *S. aureus* isolates. L, DNA Ladder; C, positive control.

The type III CRISPR-cas system was found in all 40 confirmed *S. aureus* isolates via PCR targeting the *Cas1*, *Cas2*, and *Cas10* genes, as shown in Figure 3. 

### 2.5. Identification of Spacers and Analysis of Chicken Meat Isolates of S. aureus

Online software (CRISPR-Finder) revealed that the CRISPR array had a conserved 36 bp DR sequence, 5′-GATCGATAACTACCCCGAATAACAGGGGACGAGAAT-3′, separated by 33–40 bp spacer sequences. Fifteen spacer sequences were found in all four sequenced isolates. These spacers were subjected to a BLAST nucleotide search. All sequences showed homology with the CRISPR region of various *S. aureus* strains (shown in Table 2). Based on bioinformatic analysis, we can say that isolates with the CRISPR-cas system also carry ARGs and may be associated with antibiotic resistance.

### 2.6. Expression of CRISPR-Cas Genes in Standard and MDR Strains Exposed to Antimicrobials

**Figure 4:**
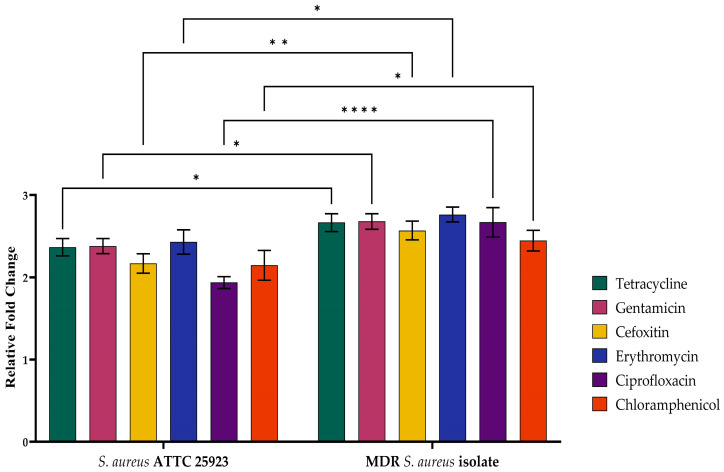
Clustered regularly interspaced short palindromic repeat (CRISPR)-cas gene expression in *S. aureus* ATCC 25923 and *S. aureus* isolates against six antimicrobials. The level of significance in pair wise multiple comparison test among the groups is presented as * (*p* = 0.0332), ** (*p* = 0.0021) and **** (*p* = 0.0001).

The interplay of the CRISPR-cas system with antimicrobial resistance was determined using qRT-PCR. There was an increased expression of the *Cas10* gene in *S. aureus* ATCC25923, and the MDR *S. aureus* isolates are presented in Figure 4. Two-way ANOVA of the relative fold change of cas10 gene expression in both *S. aureus* ATCC25923 and MDR *S. aureus* isolates against various antibiotics revealed distinctive patterns. The ATTC isolate exhibited a mean relative fold change of 2.17 ± 0.068 for the antibiotic Cefoxitin, while the MDR isolate showed a significantly higher value of 2.57 ± 0.0657. Against chloramphenicol, both *S. aureus* ATCC25923 and MDR *S. aureus* isolates displayed comparable (*p* < 0.05) mean values of 2.15 ± 0.105 and 2.45 ± 0.0724, respectively. Notably, against Ciprofloxacin, the *S. aureus* ATCC25923 strain exhibited a mean relative fold change of 1.94 ± 0.0424, which was significantly lower (*p* < 0.05) than that of the MDR isolate at 2.67 ± 0.104. Similarly, the MDR isolate revealed the highest fold expression (2.77 ± 0.0522) against erythromycin compared with that of the *S. aureus* ATCC25923 strain (2.43 ± 0.0854) and all other antibiotics. Both isolates exhibited similar (*p* > 0.05) responses to Gentamicin (*S. aureus* ATCC25923 strain: 2.38 ± 0.0525; MDR isolate: 2.68 ± 0.0547) and Tetracycline (*S. aureus* ATCC25923 strain: 2.37 ± 0.0601; MDR isolate: 2.67 ± 0.0627). 

A possible reason for increased gene expression could be because the CRISPR system is involved in regulating various genes that have an important function in membrane integrity maintenance and overcoming different stresses like antimicrobial resistance.

## 3. Discussion

The wide distribution of drug-resistant microorganisms has emerged as one of the world’s most daunting issues nowadays [25]. The use of antibiotics worldwide increased by 65% between 2000 and 2015, especially in low- and middle-income countries [26]. The rising number of reports of AMR *S. aureus* linked to food-producing animals, like poultry, has motivated surveillance research concentrating on AMR profile detection and evaluation [6]. The CRISPR-cas system, defined as a bacterial immune system, also has some other functions such as enhancing bacterial virulence [24,27,28]. The role of said system in AMR has not been discussed. Therefore, the current study was designed to assess the CRISPR-cas system’s involvement in MDR *S. aureus* through an analysis of phenotypical and genotypical processes, and bioinformatic analysis. Our investigations showed that the CRISPR-cas system plays a role in AMR, and these findings are similar to those from previously reported literature [24,29].

The present study used a conventional method and species-specific PCR to identify *S. aureus* according to a previously described method [2]. In the present study, 54.79% (40/73) of samples were found to be positive for *S. aureus* through PCR. The findings of the present study are in accordance with those of previously described studies [2,6]. 

Antibiotics are mainly used in poultry as disease prevention without any prescription, which leads to antibiotic resistance, and contaminated meat is one of the most common means of transmission of antibiotic-resistant strains of *S. aureus* to the human population. Drug-resistant pathogens are emerging as a main public health issue [30,31]. All 40 genotypically confirmed *S. aureus* isolates were analyzed to determine the AMR profile. The findings of this study showed that all isolates were resistant to erythromycin, in accordance with a previous study where 100% resistance was reported [32,33]. Resistance to tetracycline and methicillin was 97.5% and 75%, respectively, in agreement with a previous study [34]. The intermediate level of resistance estimated against ciprofloxacin is consistent with that in the previous literature [35,36], as is and the resistance against chloramphenicol [6]. This high multi-drug resistance potential might result from the uncontrolled use of antibiotics in poultry feed for disease prevention and growth [31]. 

The present study identified antibiotic-resistance genes in *S. aureus* using specific primers. PCR was used to identify the *mecA* gene, which was discussed in a previous study [36], and the *tetM* gene was identified according to the method of an earlier documented study [34]. Our findings are supported by published research reporting a 72.7% *tetM* detection rate [37]. The widespread and continued use of the same antimicrobials may be the reason for the distribution of a specific resistance gene in a region [16]. In the current study, 27% of molecularly confirmed isolates had the *ermA* gene, and 20% had the *gyrA* gene, similar to a previous study [30]. 

In this study, the CRISPR system was identified among *S. aureus* isolates with the help of PCR. It was found that all *S. aureus* isolates with antibiotic resistance were positive for the CRISPR-cas system, and this finding was in line with previous research [20,38]. However, it was also observed that the Type III CRISPR-cas system was also found in all sequenced isolates; this is not in accordance with a bioinformatic study showing that MDR Enterococci lack a CRISPR-cas system [39].

With the participation of cas proteins, the CRISPR-cas system can integrate spacers derived from foreign genetic elements. The specificity of the CRISPR-cas immune response is determined by spacer sequences [40]. Bioinformatic analysis revealed spacers among all sequenced MDR *S. aureus* poultry meat isolates. Various *S. aureus* strains displayed a resemblance with to these detected spacer sequences, and similarity was observed with these strains’ CRISPR regions. The current study’s findings are consistent with the results of earlier investigations, which discovered spacers’ similarity in the genomic component of C. jejuni strains, with their mostly resembling spacer-bearing strains [24].

This work applied qRT-PCR to assess the relationship between antibiotic resistance and the CRISPR system. The expression of CRISPR genes was higher in MDR chicken meat isolates of *S. aureus* than in the standard strain, indicating that the system is linked to antibiotic resistance. As previously stated, this enhanced expression of CRISPR genes might be due to the fact that this system regulates multiple genes having an important role in membrane integrity and resistance to various membrane stressors, like antibiotics [14,41]. Finally, in addition to defending against foreign invaders, this system mechanism contributes to antibiotic resistance. Furthermore, significant antibiotic resistance and the homology of spacers in the CRISPR region in poultry with other strains of *S. aureus* suggests that this system is involved in the development of antibiotic resistance.

Limitations of the study:

The current investigation provides a basis for a further exploration of the correlation between AMR and the CRISPR system. It is believed that further deeper study is needed to reveal whether said system enhances antibiotic resistance alone or controls numerous other genes

## 4. Materials and Methods

### 4.1. Bacterial Isolation and Growth Conditions

In this cross-sectional study, samples (N = 188) were collected from September 2022 to February 2023 from chicken meat being sold at poultry markets (Tollinton and Sheranwala) in Lahore, with sterile swabs used to vigorously swab the surface of the meat [42]. Samples were rehydrated with sterile peptone water (0.1% solution) [43], placed in an ice bag, and transported to the laboratory of the Institute of Microbiology, University of Veterinary and Animal Sciences, Lahore, Pakistan.

Samples were enriched in tryptic soy broth (TSB) and incubated overnight [9]. Each approximately 10 µL sample [44] was streaked onto Mannitol Salt Agar (MSA) and incubated for 24–48 hours [42]. Colonies with a yellow zone were sub-cultured for purification in MSA after incubation to distinguish them from other *Staphylococcus* spp. Later, these isolates were confirmed via conventional bacteriological identification methods like staining, an oxidase test, coagulase test, catalase test, IMVIC test (Indole test, M for Methyl Red test, V for Voges-Proskauer test, and C for Citrate test), urease test or citrate utilization test [45,46]. These isolates, having 20 percent *v*/*v* glycerol at −20 °C, were stored in broth until further use [47].

### 4.2. Identification of S. aureus Isolates

All presumptive *S. aureus* isolates were subjected to DNA extraction through a commercially available bacterial DNA isolation kit (Qiagen, Shenzhen, China), as described earlier [48]. Molecular species identification was performed through PCR [2] using species-specific primers as discussed in Table 3. The PCR reaction mixture (25 µL) consisted of 12.5 µL of master mix (ABM, Richmond, BC, Canada), 6.5 µL of nuclease-water, 2 µL of each primer, and 2 µL (109.8 ng/µL) of DNA template. The reaction mixture was placed in a C1000^TM^ thermocycler (Bio-Rad, Hercules, CA, USA). The thermocycling conditions were as follows: primary denaturation at 94 °C for 5 min, 30 cycles consisting of denaturation at 94 °C, annealing at 55 °C for 1 min, and extension at 72 °C for 2 min, with final extension at 72 °C for 10 min. After amplification, PCR products were separated and detected via gel electrophoresis on 1.5% agarose gel. Gel was visualized using a gel documentation system (Omega Fluor ^plus^ system, Aplegen Inc., Pleasanton, CA, USA) and GeneRuler^TM^ 100 bp Plus DNA Ladder. 

### 4.3. Antibiotic Resistance Profiling

All isolates of *S. aureus* confirmed via PCR were tested for their antimicrobial resistance using the Kirby–Bauer disc diffusion method [9]. Susceptibility tests for TE (30 µg), CN (10 µg), FOX (30 µg), E (15 µg), CIP (5 µg), VAN (30 µg), and CHL (30 µg) were performed on a Muller-Hinton Agar (MHA) plate. The diameter of the zone of inhibition was measured after overnight incubation. Cefoxitin was used for the detection of methicillin-resistant isolates. The isolates were interpreted as sensitive, intermediate, or resistant according to the Clinical and Laboratory Standard Institute [49]. MDR is considered resistant to at least one agent in three or more antibiotic classes.

### 4.4. Detection of Antibiotic Resistance Genes

Antibiotic resistance genes, like the methicillin-resistant gene (*mecA*), tetracycline-resistant gene (*tetM*), erythromycin-resistant gene (*ermA*), and quinolone resistance gene (*gyrA*), of *S. aureus* among the isolates were screened via PCR [9,16,50]. In total, 25 µl of the PCR mixture was prepared, and after the amplification of the resistance genes, PCR products were subjected to gel electrophoresis for 30 min along with a ladder (GeneRuler^TM^ 100 bp plus DNA ladder) and observed under a gel documentation system (Omega Flour ^plus^).

### 4.5. Detection of CRISPR-Cas System

The type III CRISPR system in the MDR isolates was detected using the PCR technique. Primers were used to detect the cas1, cas2, and cas10 genes. Primers were designed using genome sequences from CRISPR-positive *S. aureus* deposited in GenBank, NCBI [44]. A PCR reaction was performed, and amplified products were visualized in a gel documentation system (Omega Fluor ^plus^ system, Aplegen Inc., CA, USA). Afterward, PCR found MDR bacteria and cas10 genes, which were subjected to sequencing by Advance Bioscience International, Pakistan.

### 4.6. Detection of CRISPR Spacers

The CRISPR-cas gene sequence was subjected to a BLAST search against the reported nucleotide in GenBank. CRISPR-Finder was used to determine the sequence of CRISPR loci (https://crispr.i2bc.paris-saclay.fr/Server/, accessed on 16 June 2023). The CRISPR-Finder outputs provided the spacers from CRISPR arrays. After that, every spacer was analyzed for identification via a nucleotide BLAST search [50]. The sequence identity and *E*-values were used as alignment search criteria. The alignment’s significance was measured using an *E*-value of 0.02 as a cutoff due to the smaller spacer length and extensive database [24]. All isolates were searched, and alignments having a similarity of more than 80% and an E-value below the cutoff value were selected

### 4.7. Determination of CRISPR Gene Expression via qRT-PCR after Exposure to Antibiotics 

*S. aureus* ATTC 25923 isolates and one *S. aureus* isolate (1/2MPC) were exposed to six antibiotics of different classes. The RNA_prep_ bacterial kit (Tiagen Biotech, Beijing, China) was employed to extract RNA from each sample. To make cDNA, a one-step qRT-PCR kit (thermos-scientific) was used.

For amplification purposes, real-time PCR was performed using the CFX96 thermocycler (Bio-Rad, CA, USA). Cyclic conditions were as follows: preincubation for 3 min at 95 °C followed by 45 cycles of 10 sec at 95 °C and 40 sec at 52 °C. The primers applied were cas10*F (GTTGTACGTGCCGACATTGA) and cas10*R (GAACATCCCAACACCAGCAG). 16S rRNA, as a housekeeping gene, was utilized as an internal control for normalization. The process was carried out thrice to find the mean fold expression.

### 4.8. Statistical Analysis

The analysis was performed using SPSS™ software version 25.0 (IBM Corp., Armonk, NY, USA). The results of the resistance patterns of the samples against antimicrobials are presented as descriptive statistics in relative percentages. The relative fold expression of the cas 10 gene in both *S. aureus* ATTC 25923 and *S. aureus* isolates against various antibiotics was compared using a two-way ANOVA followed by Tukey’s post hoc test. A graphical representation of the comparison was generated using GraphPad Prism 9.5.0. (San Diego, CA, USA).

## 5. Conclusions

The study indicates a link between the CRISPR-cas system and antibiotic resistance in *S. aureus* poultry isolates. The presence of spacers in the CRISPR loci of *S. aureus* strains suggests the involvement of this system in antibiotic resistance. This implies a potential mechanism where the CRISPR-cas system plays a role in acquiring and defending against antibiotic resistance genes, emphasizing its significance in bacterial adaptation and resistance development in poultry-associated *S. aureus*.

## Figures and Tables

**Table 1:** Antibiotic resistance against various antibiotics determined via a Kirby–Baur diffusion test.

Antibiotic	Disk Concentration (µg)	Antibiotic Resistance Profile		
		*n* = 40		
		Susceptible (%)	Intermediate (%)	Resistant (%)
CHL	30	47.5	10	42.5
CIP	5	35	15	50
CN	10	65	7.5	27.5
E	15	0	0	100
FOX	30	10	15	75
TE	30	0	0	97.5
VAN	30	35	10	55

*n*, number of isolates; CHL, chloramphenicol; CN, gentamicin; CIP, ciprofloxacin; FOX, cefoxitin; E, erythromycin; TE, tetracycline; VAN, vancomycin.

**Table 2:** Spacers sequence homology of MDR *S. aureus* toother strains.

	Homology to Various Strains						
Poultry Isolates	+*S. aureus* 08BA02176 (%)	+*S. aureus* AR-0470 (%)	+*S. aureus* 110900 (%)	+*S. aureus* AR-0473 (%)	+*S. aureus* WH39 (%)	+*S. aureus* JS395 (%)	+*S. aureus* AR-0472 (%)
S. A 1	99.74	98.9	-	99.74	-	99.74	99.74
S. A 6	98.43	99	-	-	98.43	98.43	-
S. A 12	-	-	98.49	98.66	-	-	98.66
S.A 26	99.6	-	99.6	-	99.6	-	-

S. A 1, 6, 12, and 26 represent the *S. aureus* isolates in the current study; + indicates homology to the CRISPR strain’s region; indicates non-significant.

**Table 3:** Primers were used in the study to identify species-specific ARGs, and CRISPR genes of *S. aureus* isolated from chicken meat.

Target Gene	Primers (5′……3′)	Annealing Temperature	Amplicon (bp)	Reference
*Nuc*	F: AGTATATAGTGCAACTTCAACTAAA	55	450	[2]
	R: ATCAGCGTTGTCTTCGCTCCAAATA			
*mecA*	F: AAAATCGATGGTAAAGGTTGGC	55	532	[5]
	R: AGTTCTGCAGTACCGGATTTGC			
*tetM*	F: AGTGGAGCGATTACAGAA	55	158	[15]
	R: CATATGTCCTGGCGTGTCTA			
*gyrA*	F: AATGAACAAGGTATGACACC	55	223	[8]
	R: TACGCGCTTCAGTATAACGC			
*ermA*	F: AAGCGGTAAACCCCTCTGA	55	190	[27]
	R: TTCGCAAATCCCTTCTCAAC			
*Cas1*	F: GCACTCTCCATTAACGCAACT	54	432	This study
	R: AGGGGTGTTTTCTTCATAGCA			
*Cas2*	F: ACGAGAGGTATGTCAGCGAT	54	169	This study
	R: GGTTCTTTTCGCACAACAACC			
*Cas10*	F: AGAAGAGGGCGACGAAGAAT	54	500	This study
	R: ACTGCTGACATTACGCCAAA			

## Data Availability

All data are available in the manuscript.
